# Current practice of epidemiology in Africa: highlights of the 3^rd^ conference of the African epidemiological association and 1^st^ conference of the Cameroon society of epidemiology, Yaoundé, Cameroon, 2014

**DOI:** 10.11604/pamj.2015.21.256.7556

**Published:** 2015-08-07

**Authors:** Armand Seraphin Nkwescheu, Joseph Fokam, Patrice Tchendjou, Akindeh Nji, Hermann Ngouakam, Bita Fouda Andre, Sobngwi Joelle, Benjamin Uzochukwu, Kingsley Akinroye, Wilfred Mbacham, Vittorio Colizzi, Rose Leke, Cesar Victora

**Affiliations:** 1 Cameroon Society of Epidemiology (CaSE), Yaoundé, Cameroon; 2 Division of Health Operations Research (DROS), Ministry of Public Health, Yaoundé, Cameroon; 3 Laboratory of Public Health Biotechnology (LPHB), University of Yaoundé I, Yaoundé, Cameroon; 4 African Epidemiological Association (AEA), Abuja, Nigeria; 5 Faculty of Medicine and Biomedical Sciences, University of Yaoundé I, Yaoundé, Cameroon; 6 Chantal BIYA International Reference Centre (CIRCB) for research on HIV/AIDS prevention and management, Yaoundé, Cameroon; 7 University of Rome Tor Vergata, Rome, Italy; 8 Epidemiology and Public Health Service, Centre Pasteur du Cameroun (CPC), Yaoundé, Cameroon; 9 Faculty of Sciences, University of Yaoundé I, Yaoundé, Cameroon; 10 African Synergies against AIDS and Suffering, Yaoundé, Cameroon; 11 Faculty of Medicine and Pharmaceutical Sciences, University of Douala, Cameroon; 12 Ministry of Public Health of Cameroon; 13 Catholic University of Central Africa (UCAC), Yaoundé, Cameroon; 14 University of Nigeria, Enugu Campus, Nigeria; 15 Nigerian Heart Foundation, Lagos; 16 International Epidemiological Association, Brazil; 17 Universidade Federal de Pelotas, Departamento de Medicina Social Pelotas, RS, Brazil

**Keywords:** Highlights, 3rd epidemiology Conference, Africa, Yaoundé, Cameroon

## Abstract

As the study of disease occurrence and health indicators in human populations, Epidemiology is a dynamic field that evolves with time and geographical context. In order to update African health workers on current epidemiological practices and to draw awareness of early career epidemiologists on concepts and opportunities in the field, the 3^rd^ African Epidemiology Association and the 1st Cameroon Society of Epidemiology Conference was organized in June 2-6, 2014 at the Yaoundé Mont Febe Hotel, in Cameroon. Under the theme**«Practice of Epidemiology in Africa: Stakes, Challenges and Perspectives»**, the conference attracted close to five hundred guest and participants from all continents. The two main programs were the pre-conference course for capacity building of African Early Career epidemiologists, and the conference itself, providing a forum for scientific exchanges on recent epidemiological concepts, encouraging the use of epidemiological methods in studying large disease burden and neglected tropical diseases; and highlighting existing opportunities.

## Introduction

The concept and practice of Epidemiology, known as the study of disease occurrence and health indicators in human populations, is evolving globally and requires updates for practitioners in the field [1, 2]. Of note, the African region of the World Health Organization (WHO/Afro) supports forum aimed at reducing gaps in epidemiological production [3]. Under the auspices of the International Epidemiological Association (IEA), some epidemiologists met in Calabar (Nigeria) in March 2012 to create the African Epidemiological Association (AEA), which represents the federation of national associations of epidemiology within the forty-six Member states of WHO/AFRO [4]. A key objective of AEA is to promote the discipline of epidemiology in the African continent, through a yearly conference in order to sustain and promote interactions, research and exchange of knowledge between epidemiology practitioners [4]. After its second conference in Abuja in 2013, it was deemed necessary to design a special conference that will respond to specific needs of African epidemiologists.

## Conference outlines and outcomes

The present conference was the third for AEA and the first for the Cameroon Society of Epidemiology (CaSE), organized from June 2-6, 2014 at the Mont Febe Hotel in Cameroon, entitled **«Practice of Epidemiology in Africa: Stakes, Challenges and Perspectives»**, and organized into two main programs: (1) a pre-conference course on Epidemiology and (2) a conference structured into symposia, plenary sessions, oral and poster sessions.

**Preconference course:** this was a two-day course on capacity-building on Epidemiological methods held from Monday 2^nd^ to Tuesday 3^rd^ June 2014, brought to light (by lessons) the **Practice of Epidemiology for Early Career Epidemiologists in Africa**, delivered by faculty members from renown universities in Europe (France) and Africa (South-Africa, Nigeria and Cameroon). This fully booked course, attended by more than a hundred participants, provided basic and practical concepts in Epidemiology as well as training and job opportunities.

**The conference:** this scientific forum held from Wednesday 04th to Friday 06th June 2014, with the three main objectives, which were: (a) to provide a forum for scientific exchanges on recent advances in epidemiology; (b) to encourage the use of epidemiological methods in the study of large disease burden as well as neglected tropical diseases; and (c) to promote Epidemiology and highlight existing opportunities. This event attracted closed to five hundred guests and participants from Brazil, Canada, France, USA, and African countries (Nigeria, Malawi, South-Africa, Ghana, Kenya, Uganda, Democratic Republic of Congo, and Cameroon). The program covered Infectious diseases, Non-communicable diseases, Neglected tropical diseases and Health systems in Africa, and was structured under plenary sessions, symposia, workshops, oral/poster presentations, exhibitions, and the AEA general assembly.

**Plenary sessions:** key lessons from plenaries were the inaugural discourse by Cesar G. Victora (IEA President) on **"Epidemiology and the Millennium Development Goals (MDGs): Lessons for the post- 2015 Agenda"**, with special focus on MDG1 (reduce by 50% the prevalence of pediatric underweight), MDG4 (reduce under-five mortality by 2/3), MDG5 (reduce maternal mortality by 75%) and MDG6 (control HIV/AIDS, TB, malaria, other infections). Roles of stakeholders, tracking progress, evidence-based actions and accountability appeared very important in disseminating country-level data, equity, policy, health systems reforms, financing, and tracking indicators across the continuum of care at country and global levels in the post-2015 era. These health challenges were in line with statements of the Minister of Public Health, Andre Mama Fouda (alongside several stakeholders including WHO-country Representative), who underscored the relevance of epidemiology in disease surveillance and control in African health programs, as well as the presentation of the Conference Scientific committee Chairperson (Prof. Rose Leke) encouraging the translational application of conference knowledge into application in respective health programs and research in epidemiology.

**Sessions on infectious diseases:** this mainly covered infectious diseases of public health importance, among which HIV/AIDS, Malaria and Tuberculosis. Regarding **HIV/AIDS**, updated guidelines on antiretroviral therapy (i.e. starting treatment at CD4 < 500 cell/mm^3^, importance of viral load measurement) and research interests (third line regimens, clinical trials and HIV functional cure). Improved community awareness on HIV/AIDS related Knowledge, attitudes and practices are needed, including condom use and stigmatization. Patients receiving ART appeared unable to subsidize their treatment costs. PMTCT strategies (with exclusive maternal breastfeeding and clinic attendance) showed promising outcomes, while the implementation of family planning in African HIV programs will favor safe contraception for HIV-infected women. However, sustaining HIV-1 Early Infant Diagnosis program remains challenging and requires local measures for a successful PMTCT performance, including maternal non-return to clinic attendance, socio-economical and poor ANC profile (recommending maternal ANC education and cost subsidy). ART performance was successful due to ongoing free drug dispensing and counseling; stock-outs of antiretrovirals, poor adherence and community disengagement are still a concern. Current treatment of Hepatitis C and B were presented, major challenges being drug unaffordability. Regarding **Malaria parasite**, recent findings highlighted products effectiveness prior to public health utility, community sensitization for maximum compliance, improved and sustained health education for better malaria management, the importance in training patent medicine vendors on national malaria guidelines and strengthening home management of malaria. Reliability of rapid diagnostic tests (RDT), performance of ITNs and challenges in malaria vaccine research were addressed, including indoor residual spraying, women empowerment in pregnancy management and malaria treatment and control, treatment, resistance surveillance, and funding restrictions. There was also a presentation on data needed on T and B cell responses in pregnancy. Researchers working on Malaria were encouraged to join MIM (Multi-Initiative for Malaria) for newsletter sharing. Regarding **Mycobacterium Tuberculosis**, the efficacy of Quantiferon-TB for latent MTB diagnosis showed specific antigenic peptides among Cameroonian children. For TB, String test was shown to reduce the turn-around-time and to favor testing acceptance; meanwhile Gene Xpert MTB/RIF assay was proven useful for TB treatment. Effective treatment and prevention of MDR-TB require compliance and public awareness.

**Session on non-communicable diseases** addressed task shifting in low- and middle-income countries, using diabetes as an example to improve community healthcare with non-professional health workers (NPHWs), staff retention, irregular drug supply and limited equipment. The WHO-stepwise approach for the global strategy for the prevention and surveillance of NCDs is a priority, especially for type-2 diabetes that is the most prevailing. Increasing CVD and strokes in Africa are due to high tobacco and alcohol consumption, suggesting tobacco surveillance. The high cancer burden in Africa (breast, cervix, uterus, liver, Burkitt lymphoma), with a 1.4 million prediction by 2030 from 715,000 in 2003, requires local response. EBV significantly interacts with malaria in endemic settings.

**Session on Neglected Tropical Diseases (NTDs):** interests centered around high burden of NTDs in Africa (14/17 NTDs for 1.4 billion inhabitants), impact of climate change on health, R&D capacity, technology transfer and elimination of poverty-related NTDs. For Buruli ulcer (BU), lack of appropriate diagnosis, limited therapeutic protocols, limited evidences on transmission routes and on determinants of HIV/BU co-infection are serious concerns. Lymphatic filariasis warrants reliable diagnosis and prevention of drug adverse events.

**Session on health Systems** addressed strategies for improving health systems in Africa through systematic reviews, enhancing values of individual studies, using Cochrane reviews as example. Health policy and systems research & analysis (HPSR+A) in poor settings showed poor interventions, requiring human resources, governance, service delivery, financing, medical data and technology). With examples from Profs. Uzochukwu and Shey, HPSR+A could be considered as an “*emerging* *field that seeks to understand and improve how societies organize themselves in achieving collective health goals, and how different actors interact in the policy and implementation processes to contribute to policy outcomes.”*..Governmental involvement is an essential component for evidence-based policy-making.

**Symposia sessions** addressed innovative collection and stabilization methodologies for greater accessibility and transportation of infectious disease samples (by Jiravatvisanuvimol, DNA Genotek inc., Ottawa, Canada); and on Skills and Core Competency of an Epidemiologist (by Debashis Basu, RSA), mainly targeting early career epidemiologists.

**Poster session:** 50 of 110 accepted abstracts were presented, with 49% (24/49) displayed in Day-1 covering dental care, cosmetic-related dermatology, health insurance, occupational health, trans-generation relationship, food hygiene, palliative care, anti-infectious agents, performance of rapid testing, PMTCT, reproductive health, behavioral sciences, immunization programmes, outbreaks and epidemiological surveillance, health policies, waste management, hospital hygiene, nutrition, NCDs and cancer. 43% (26/61) were displayed in Day-2, covering NTD, management of rabies virus, depression, HIV and HBV, helminths, herpes viruses, malaria co-infections, as other co-infections; knowledge, attitudes and practices studies; performance of malaria rapid diagnosis tests, antimalarial agents, NCDs, zoonosis, gastro-enteritis, polio vaccination, cholera and surveillance, epidemiology, TB and vector control strategies.

**AEA General Assembly Meeting (AGM):** chaired by AEA President (Dr Kingsley Kola Akinroye), experienced a growing number of membership countries (148 members: 111 in West Africa, 27 in Southern-Africa and 10 in East-Africa), with a need to invite Northern Africa. The African Journal of Epidemiology was presented by Chief-Editor (Prof. Margaret Araoye) and calling for publication of the conference presentations. The 2014 World Epidemiology Congress was announced for August 2014 in Alaska, and the 2017 World congress in Japan; Africa will bid for the host of the 2020 World Epidemiology Congress, and applications are welcomed. Exhibitions were dominated by stands among which were the CaSE, Medecins Sans Frontieres (MSF), Universite des Montagnes, and other national agencies.

Summary of covered program (by Dr. Joseph Fokam), as well as addresses by the Scientific Chairperson (Prof. Rose Leke), the AEA-representative (Dr. Debashis Basu), and Chairperson of CaSE (Dr. Armand S. Nkwescheu), marked the end of the conference.

## Conclusion

This conference was of great asset to expose young career epidemiologists in the practice, training opportunities and research related to the field. International participation provided opportunities for research collaboration in public health and epidemiology, as well as perspectives in the field of epidemiology and public health.
